# Evaluating the Impact of Authentic Research on Secondary Student Self-efficacy and Future Scientific Possible Selves

**Published:** 2018-11-12

**Authors:** Naomi Delaloye, Lisa Blank, Desirae Ware, Carolyn Hester, Tony Ward, Andrij Holian, Earle Adams

**Affiliations:** 1 University of Montana, USA

**Keywords:** NGSS, possible scientific selves, self-efficacy, secondary science

## Abstract

**Background:**

As the need to involve more students in STEM learning and future careers becomes more pressing, identifying successful methods of engaging students in meaningful scientific learning that increases their interest in science is essential. Student self-efficacy (their confidence or belief in their ability to accomplish tasks) is closely tied to student interest in science, as is student future scientific possible selves.

**Material and Methods:**

This manuscript presents the findings of a study that evaluated the Clean Air and Healthy Homes Program (CAHHP), which provides students the opportunity to design and implement authentic scientific research on indoor air quality issues. The program’s influence on student self-efficacy, scientific research and experimental design skills, and future scientific possible selves was examined. Students (n=169) from six schools completed a pre- and post-assessment at the beginning and end of the program.

**Results:**

Results showed the greatest impact on student research self-efficacy, along with improvement in student research and experimental design skills.

**Conclusions:**

We conclude that programs promoting authentic learning opportunities aligned with the most recent national science standards show great promise in improving both student interest and skills in science.

## INTRODUCTION

The need for students to pursue careers in science, technology, engineering, and mathematics (STEM) is at the forefront of today’s educational discourse (Forum, 2010; National Science and Technology Council, 2013). The recognition of this need has led to a growing body of research focused on the factors influencing student interest in science as a career (Potvin & Hasni, 2014). It is assumed that students with strong science skills will succeed in science classes and therefore consider careers in science. However, a report from the Business-Higher Education Forum (2010) indicates that there is a more nuanced reality. They found that a significant portion of secondary students (25.4%) have a high proficiency in science, but low interest. Their results further showed that 15.2% of students had low proficiency, but high interest in science. It is therefore critical that science educators explore how they can increase interest in science for these skilled but low-interest students, and how skills can be improved for interested, but lower-performing students.

Extensive research has examined how *teaching methods* affect student learning (Swarat, Ortony, & Revelle, 2012). However, this effort has provided only a limited understanding of successful science education practices. In response, a growing body of research highlights how affective, or emotional, factors influence whether or not a student chooses a career in STEM (Adedokun, Bessenbacher, Parker, Kirkham, & Burgess, 2013; Potvin & Hasni, 2014; Schunk & Pajares, 2002). The most discussed of these is self-efficacy, defined as one’s belief in their ability to learn and perform tasks within a subject area. Building on the work of Albert Bandura (1986, 1997), Lent, Brown, and Hackett (1994) set forth the social cognitive theory of career development in which they describe a number of propositions about how self-efficacy affects career choice, including:

*Proposition 1*: An individual’s occupational or academic interests at any point in time are reflective of his or her concurrent self-efficacy beliefs and outcome expectations;

and

Proposition 2: An individual’s occupational interests also are influenced by his or her occupationally relevant abilities, but this relation is mediated by one’s self-efficacy beliefs.

(p. 91 and 92).

These propositions suggest that a student’s career interests at any time are directly influenced by his/her belief in their ability in that area, and that skills in the area also influence career choice. This highlights the relationship between scientific attitudes and skills. The two elements of learning feed one another and both should be considered when determining *what* is learned in the science classroom and *how*.

Another aspect of social cognitive theory is the concept of possible selves, defined as “a person’s ideas of what one would like to become and what one is afraid of becoming” (Hong & Greene, 2011). Future *scientific* possible selves are individuals’ views of their future selves as scientists. A number of studies have examined influences on students’ possible selves, which include: mentorship (Nugent et al., 2015; Packard & Nguyen, 2003), opportunities in informal learning environments (Mills, 2014), and their connection to students’ social identities (Oyserman, Bybee, & Terry, 2006). However, there is still room for growth in understanding how the everyday activities of the science classroom affect students’ future scientific selves.

With the development and implementation of the new *Next Generation Science Standards (NRC, 2013)*, science teachers are being asked to reevaluate their teaching methods and activities. The new *Framework for K-12 Science Education: Practices, Crosscutting Concepts, and Core Ideas* set forth by the National Research Council (2012) attempts to shift national attention from high-stakes, rote learning, to a more authentic form of science education. The goal of the framework is that “by the end of the 12^th^ grade, students should have gained sufficient knowledge of the practice, crosscutting concepts, and core ideas of science and engineering to engage in public discussions on science-related issues, to be critical consumers of scientific information related to their everyday lives, and to continue to learn about science throughout their lives” (NRC, 2012). This is achieved by engaging students in learning that interweaves key content learning objectives, crosscutting concepts, and base scientific skills, or “practices”. The three-dimensional framework recognizes that students cannot simply learn discrete facts or the scientific method, but rather that students need to engage in a more complete form of science in which former knowledge informs future exploration.

One dimension of the framework, the Eight Practices (see Appendix A), focuses on *how* one does science and presents scientific information. However, the authors “are quick to emphasize that inquiry entails the fluid, integrated, and iterative interplay among [the] three dimensions of learning” (Surr, Loney, Goldston, Rasmussen, & Anderson, 2016, p. 8). Previous interpretations of scientific inquiry, as defined by the National Science Standards (NRC, 1996), were widely variable, and often boiled down to being seen as a set of steps for students to complete in order to ‘do science’. Rather than being seen as an integral part of teaching broader content, inquiry often became isolated and taught as its own content area. Through the three-dimensional model and the greater integration of the eight science practices, the NGSS aim to redefine how ‘inquiry’ is conceptualized and enacted in the classroom. As Surr et al. (2016) note, “moving scientific inquiry out of the content standards conveys the notion that inquiry should not stand alone, but rather it should be interwoven throughout all science learning” (p.10).

In theory, the creation and implementation of the *Framework for K-12 Science Education* and the *Next Generation Science Standards* provide an opportunity to engage students in meaningful, engaging science education, thereby increasing their self-efficacy and their possible scientific selves. However, as the Framework and the NGSS are relatively new, there is need to explore the effect of the learning opportunities they provide on students’ self-perceptions of themselves as scientists and whether or not they become more inspired to pursue careers in science. Do specific teaching and learning strategies that support the Framework positively influence student attitudes and career choice? Science educators must develop a clear understanding of which classroom teaching methods improve not only student learning, but also students’ scientific selves and self-efficacy as these have a profound impact on career choice. The Clean Air and Healthy Homes Program is one among many trying to address these important questions.

## THE CLEAN AIR AND HEALTHY HOMES PROGRAM

*The Clean Air and Healthy Homes Program* (CAHHP) is a science education outreach program designed to offer middle and high school students pathways to conduct authentic scientific research through the real-world issue of air quality in their homes and communities. Originally named *Air Toxics Under the Big Sky*, the program has evolved and grown significantly since its inception in 2003 (Adams et al., 2008; Marra et al., 2011). The current program has three primary objectives: 1) To develop and provide inquiry-based, learner-centered instructional materials and opportunities to secondary students; 2) to implement these learning materials in rural underserved areas; and 3) to provide professional development opportunities for teachers interested in environmental health sciences. The overarching goal is to increase student interest in pursuing a career in science through a relevant environmental science research opportunity.

Through CAHHP, students learn about three major air pollutants (particulate matter, radon, and carbon monoxide), all of which cause adverse health effects and are commonly found in indoor environments, such as homes and schools. The program takes place over the course of an entire school year, during which students explore the pollutants through a series of lesson plans and then design and implement their own scientific research projects focused on one of the pollutants. All of these activities integrate the NGSS Eight Practices and are centered around student-focused learning. Through their research on pollutants in their immediate surroundings, students begin to understand the link between their own health and potential exposures in their environment, as well as possible solutions to these problems.

Once students have designed and implemented their research, they have the opportunity to present their findings at an annual CAHHP symposium which is held on the university campus. Students present their data through either a PowerPoint or poster and explain the significance of their work. This is a very valuable element of the program as a study focused on inquiry-based science curricular initiatives developed between 1998 and 2007 found that only about 10 percent of projects emphasized presenting and communicating findings (Asay & Orgill, 2010). If students are unable to attend the symposium, they often present their work at other formal events such as regional and state science fairs.

Each school year, around 800 students from a variety of secondary classrooms including chemistry, biology, environmental science, and anatomy and physiology, participate in CAHHP and perform research on air quality issues within their homes and communities. Overall, CAHHP, with its emphasis and support of integrating the *Eight Practices* into content learning, provides a platform for engaging in authentic scientific learning opportunities encouraged by the NGSS.

In order to better understand the effect of CAHHP on student learning and attitudes, an external evaluation was completed to answer the following questions:

1. Did classes that participated in the CAHHP curriculum show increased self-efficacy in scientific research skills?
2. Did classes that participated in the CAHHP curriculum show an increase in positive possible scientific selves?
3. Did classes that participated in the CAHHP curriculum show an increase in proficiency in scientific reasoning?
4. Did classes that participated in the CAHHP curriculum show increased proficiency in experimental design?
5. Do higher scores on experimental design, scientific research and scientific reasoning skills predict higher possible scientific selves?
6. Are there differences in measured outcomes based on gender or ethnicity?

## METHOD

During the 2014/2015 school year, a pre- and post-program survey was administered to participating students (n=169) within six schools. Participating students came from seven junior and senior level, elective high school classes in communities with populations between 3,000 and 70,000. For a breakdown of student gender and ethnicity, see Table 1. Not all teachers participating in the program had their students complete the survey. Teachers whose students were participating in the survey were asked to administer a pre-survey with their students prior to implementing any of the CAHHP lesson plans and materials, typically toward the beginning of the school year during September or October (for an overview of CAHHP learning materials, see Delaloye et al., 2016). Survey questions targeted the following areas of student growth: scientific research skills self-efficacy, experimental design proficiency, scientific reasoning proficiency, and possible scientific selves. It consisted of 53 questions, which included:

- 12 Likert-scale questions related to student self-efficacy in employing research strategies in science learning.
  **Example:**
  I can analyze the results of a scientific investigation.

**Table:** 

Sometimes	Usually	Always
- 18 Likert-scale questions regarding students’ STEM career interest.
  **Example:**
  How interested are you in having a job like these someday? For each question, select the response that makes the most sense to you.
  **Doctor, Dentist, Vet**

**Table:** 

Very Interested	Somewhat Interested	Not Interested
- 14 Likert-scales questions related to students’ scientific possible selves.
  **Example:**
  The following questions explore your plans for the future. For each question, select the response that makes the most sense for you.
  People like me become scientists.

**Table:** 

Agree a lot	Agree some	Don’t agree
- 7 Selected Response questions evaluating student proficiency with experimental design.
  **Example**
  Two studies estimate the mean caffeine content of an energy drink. Each study uses the same test on a random sample of the energy drink. Study 1 uses 25 bottles, and study 2 uses 100 bottles. Which statement is true?
  - The estimate of the actual mean caffeine content from each study will be equally uncertain.
  - The uncertainty in the estimate of the actual mean caffeine content will be smaller in study 1 than study 2.
  - The uncertainty in the estimate of the actual mean caffeine content will be larger in study 1 than study 2.
  - None of the above
- One Constructed Response question to evaluate student proficiency with experimental design.
  **Example**
  Advertisements for an herbal product, ginseng, claim that it promotes endurance. To determine if the claim is fraudulent and prior to accepting this claim, what type of evidence would you want to see? Outline details of your investigative design.
- One constructed response asking students to develop a science explanation in response to the prompt below:
  **Example:**
  Consider the following data: Liquid One has a density of 0.93 g/cm3; no color, mass of 38 g, and melting point of −98 degrees Celsius. Liquid Two has a density of 0.79 g/cm3, no color, a mass of 38g, and a melting point of 26 degrees Celsius. Liquid Three has a density of 13.6 g/cm3, a silver color, a mass of 21 g, and a melting point of −39 degrees Celsius. Liquid Four has a density of 0.93 g/cm3, no color, a mass of 16 g, and a melting point of − 98 degrees Celsius.

Write a science explanation that states whether any of the liquids are the same substance.

Two demographic questions about gender and ethnicity were also included in the survey, which was administered online, taking approximately 30 minutes to complete. Teachers had students complete the same survey at the end of the year after students had designed, executed, and presented their CAHHP research projects, typically at the end of May.

Results from the survey that pertained to research questions 1 through 4 were analyzed using an independent sample *t-*test, while results from the survey pertaining to research question 5 were analyzed using a multiple linear regression. Finally, results for research question 6 were analyzed using an analysis of variance (ANOVA).

## RESULTS

**Figure 1.**
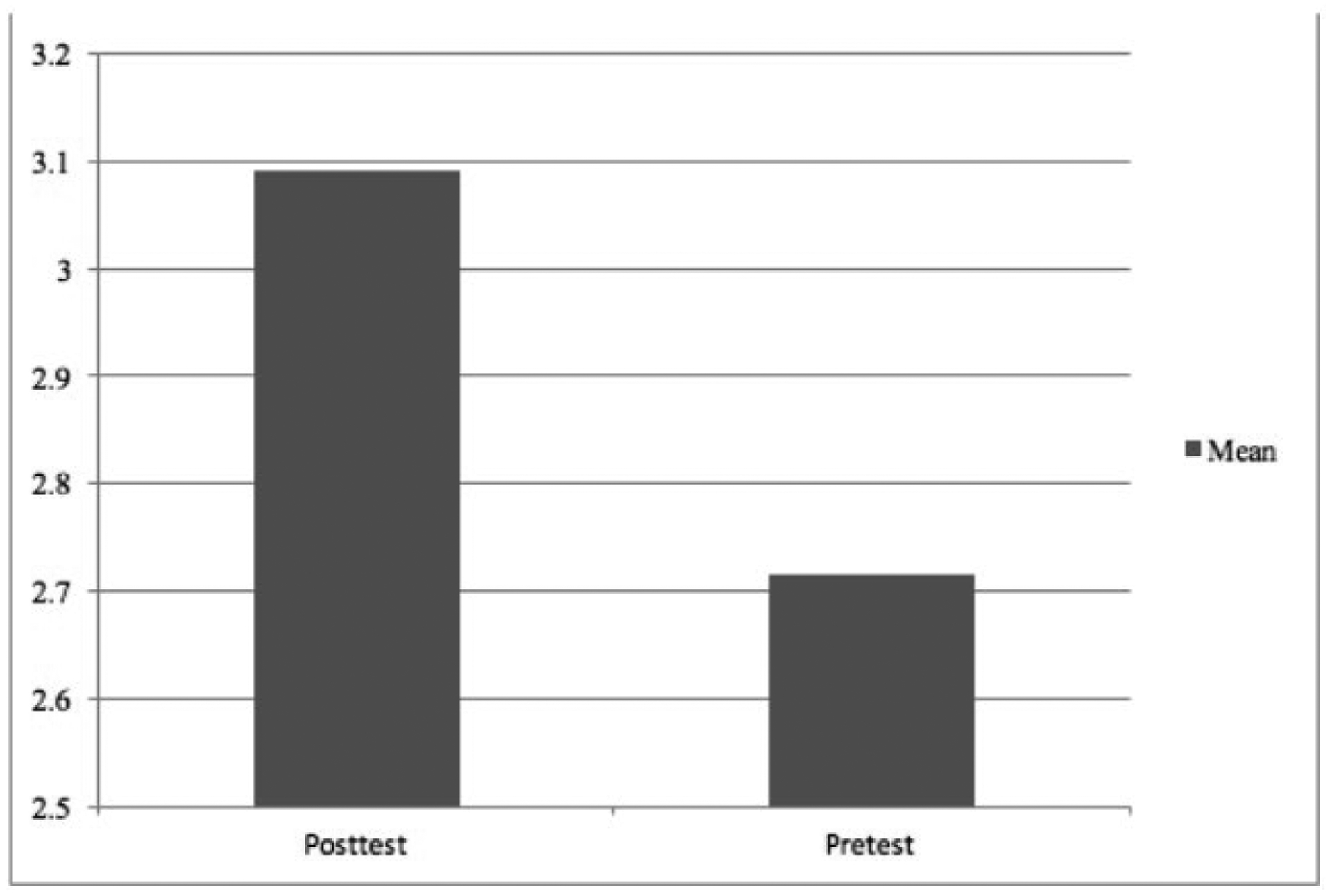
Mean scientific research skill self-efficiency pre- to post assessment

In testing whether or not classes that participated in CAHHP showed an increase in self-efficacy in scientific research skills, the results of the independent sample *t*-test were significant (*t* (276) = 4.61, *p* < .001) suggesting that student self-efficacy did increase (see Table 2). The post-test had a significantly higher mean compared to the pre-test. According to Cohen (1988), the difference between the two groups was a medium effect size. Figure 1 shows the averages of scientific research skill self-efficacy pre- to post. Table 3 shows these results disaggregated, reflecting where self-efficacy in various scientific research skills was most improved over the school year, which was primarily in areas related to data analysis and presentation of findings.

Regarding whether or not students’ positive scientific selves improved, the results of the independent sample *t*-test, presented in Table 4, were not significant, *t*(274) = −1.12, *p* = .264, suggesting that there was no difference observed in scientific possible selves from pre- to post.

Results of the independent sample *t*-test for science reasoning skills by test are presented in Table 5. Classes that participated in the program did show an increase in scientific reasoning skills as the results of the independent sample *t*-test were significant, *t*(274) = 3.36, *p* < .001. The post-test mean was significantly higher compared to the pretest mean. According to Cohen (1988), the difference between the two groups was a small effect size.

**Figure 2.**
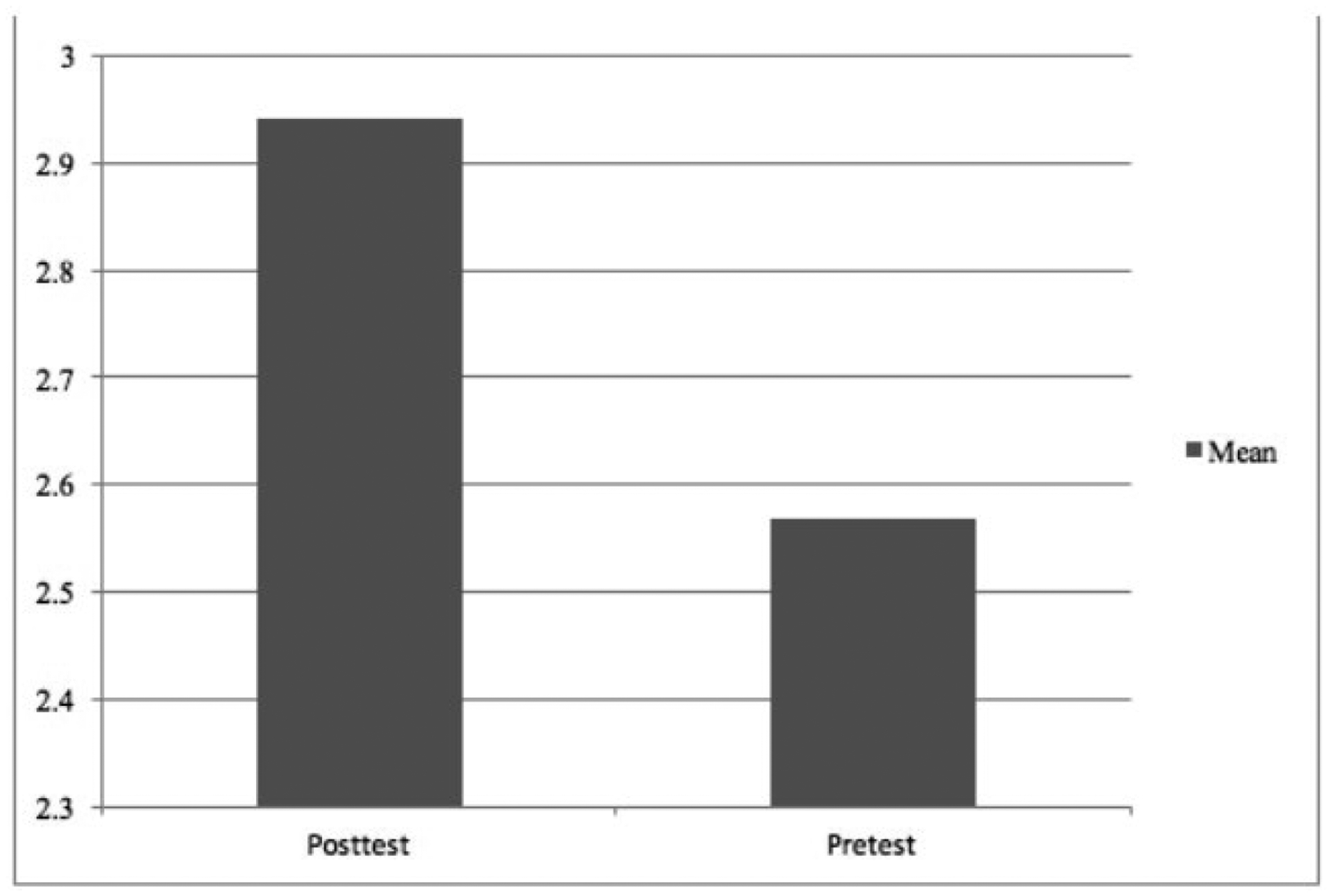
Student averages of experimental design skills pre- and post assessment

Results of the independent sample *t*-test for student experimental design proficiency are presented in Table 6, with Figure 2 showing the averages from the pre- and post-assessments. The results of the independent sample *t*-test testing were significant, *t*(276) = 2.70, *p* = .007, suggesting that students’ ability to design an experimental test increased from pre- to post assessment. The post assessment had a significantly higher mean than the pre-test.

In testing if experimental design, scientific research skills, and scientific reasoning skills were predictors of scientific possible selves, the results of the linear regression were significant, *F*(3,271) = 22.88, *p* < .001, *R*^*2*^ = 0.20, suggesting that these factors accounted for 20% of the variance in scientific possible selves (see Table 7). The individual predictors were examined further and while experimental design was not found to be a significant predictor of scientific possible selves, scientific research skills was a significant predictor of scientific possible selves (*B* = 0.35, *p* < .001). This suggests that for every one unit increase in scientific research skills, scientific possible selves increased by 0.35 units. Also, scientific reasoning was a significant predictor of scientific possible selves (*B* = 0.06, *p* = .030) suggesting that for every one unit increase in scientific reasoning, scientific possible selves increased by 0.06 units.

Regarding performance results based on ethnicity, the results of the ANOVA were significant, *F*(18, 259) = 2.04, *p* = .009, partial η^2^ = 0.12, suggesting there was a difference in scientific research skills by ethnicity. A partial η^2^ of 0.12 suggests a moderate difference between the groups. For gender, the results of the ANOVA testing showed that the difference between males and females in scientific possible selves were significant (*F*(2, 274) = 3.23, *p* = 0.041, partial η^2^ = 0.02). A partial η^2^ of 0.02 suggests a small difference between the groups with females scoring slightly higher than males. Finally, regarding scientific research skill based on gender, the results of the ANOVA were not significant (*F*(2, 275) = 0.40, *p* = 0.668, partial η^2^ = 0.00) suggesting there was no difference in research skill outcomes by gender.

## DISCUSSION

Results from this evaluation suggest that the greatest impact of the CAHHP curriculum on student outcomes is increased self-efficacy in scientific research skills. When breaking down the skills further, the curriculum was most successful in elevating students’ perceived ability to analyze data, use graphs and models to explain and display results, communicate results to others using science terms, and use results to help answer questions students have about their environment or make decisions that affect their health. These findings support the theory that self-efficacy is a dynamic set of beliefs and not static traits (Bandura, 2012). As Lent et al. (1994) describe, “In [Bandura’s] view, mastery of challenging tasks engenders positive self-evaluation; the anticipation of additional mastery and self-satisfaction helps sustain task engagement, leading to skill development, and the growth of interest in activities that may have originally held little intrinsic allure” (p. 90). These findings further highlight how, as educators, there is the possibility to positively impact students’ perceived ability in a subject, self-efficacy, thereby increasing their interest, and possibly their future career choice. If Bandura is correct, engaging students in meaningful science learning experiences and increasing their self-efficacy is a direct way to heighten interest in a science career. Other research has shown a direct effect of self-efficacy on student achievement and persistence (Schunk & Pajares, 2002). CAHHP models one type of learning program that shows promise in improving student interest in science careers by increasing student self-efficacy. This is valuable when considering the 25% of students mentioned previously who are skilled in science, but have low interest (Forum, 2010).

The program also shows promise in benefiting students who may have a high interest in science, but lower ability as the results show improvement in students’ scientific reasoning skills and experimental design. Although the increase in scientific reasoning was not as prominent as the improvement of self-efficacy, participating students primarily came from upper level, elective science classes, an indicator that they already had a relatively strong base in these areas. Overall, our evaluation results support the idea that elevated self-efficacy in research skills can help students design and conduct independent research projects and communicate scientific findings – a primary goal of the program.

Additionally, the development of these skills is noteworthy given their alignment with five of the eight science practices outlined in the NGSS: 1) asking questions, 2) developing and using models, 3) analyzing and interpreting data, 4) constructing explanations, and 5) obtaining, evaluating, and communicating information. A stated goal of the NGSS (NRC, 2012) is that “students should have gained sufficient knowledge of the practice, crosscutting concepts, and core ideas of science and engineering to engage in public discussions on science-related issues, to be critical consumers of scientific information related to their everyday lives, and to continue to learn about science through their lives” (p. 9). The evaluation results show that a program like CAHHP not only shows promise in helping students develop relevant science skills, but also aids them in becoming critical consumers of scientific information related to their everyday lives (see Table 3, Strategy 2). These results support the notion that the integration of the Eight Practices into the curriculum is an effective way to not only improve their science skills, but also meaningfully engage students in the world around them.

While no statistically significant growth was noted in scientific possible selves, it should be noted that results from the pre-survey show that participating students had very high positive scientific selves at the beginning of the school year. Again, many of the students participating were in the upper grades of high school and already had strong views of their future selves. In upcoming school years, including more students from the early high school grades in CAHHP would provide an opportunity to better analyze how open-ended research projects may or may not improve positive scientific selves. Our study was also limited by the absence of a control group, which needs to be addressed in future years in order to confirm the program’s effect.

## CONCLUSION

CAHHP shows promise in improving both self-efficacy and scientific reasoning skills. If these are indeed predictors of career choice, more students should be given the opportunity to do research from their own design that is relevant to their everyday lives. Our results suggest that using the Eight Practices of the NGSS as a vehicle for learning engages students in hands-on learning that is meaningful, and improves both student learning and attitudes. It is logical that students perform better when learning is tangible and relevant. Activities that treat science learning as multi-dimensional and dynamic, such as those encouraged by the NGSS, tap students’ natural curiosity through exploration of the world around them. Student outcomes from participating in CAHHP support this is as a valid learning method.

Results from our evaluations support the value of the current trend in science education: getting students to think like scientists, to indeed *be* scientists, by engaging in learning that *is* science. The more we successfully get students to think like scientists, the better we are preparing students of future generations to be competent and confident scientists in the workforce. As next steps in our study, we are interested in including an analysis of how participating in CAHHP affects learners in earlier secondary grades, how CAHHP improves student self-efficacy and scientific reasoning skills, and correlations with interests in science careers.

## Figures and Tables

**Table 1:** Summary of CAHHP survey participants (all self-identified)

		European American	American Indian	Asian	Multi-Racial
Male	N=69 (43%)	56%	3%	2%	39%
Female	N=100 (57%)				

**Table 2.** Independent Sample t-Test of Self-Efficacy in Scientific Research Skills

				Pretest		Posttest	
Variable	*t* (276)	*p*	Cohen’s *d*	*M*	*SD*	*M*	*SD*
Scientific Research Skills	4.61	0.001	0.58	2.71«M1»	0.69	3.09	0.61

**Table 3.** Highlighted rows indicate areas of greatest growth when students’ self-efficacy in research skills is disaggregated

Strategy 1: I can use scientific knowledge to form a question about air quality.
Strategy 2: I can use science to help me make decisions that affect my health.
Strategy 3: I can ask a question about air quality that can be answered by collecting data.
Strategy 4: I can communicate a scientific procedure that examines air quality to others.
Strategy 5: I can record air quality data accurately.
Strategy 6: I can use data to create a graph about air quality for presentation to others.
Strategy 7: I can create a display to communicate my data and observations.
Strategy 8: I can analyze the results of a scientific investigation.
Strategy 9: I can use science terms to share my results.
Strategy 10: I can use models to explain my results.
Strategy 11: I can use the results of my investigation to answer science questions I pose.
Strategy 12: I can explain which science concepts guide my research questions.

**Table 4.** Independent Sample t-Test for Future Plans (Scientific Possible Selves) Composite by Test

				Pretest		Posttest	
Variable	*t*(274)	*p*	Cohen’s *d*	*M*	*SD*	*M*	*SD*
Scientific Possible Selves	−1.12	0.326	0.13	3.94	0.81	3.85	0.46

**Table 5.** Independent sample t-test for science reasoning skills by test

				Pretest		Posttest	
Variable	*t*(274)	*p*	Cohen’s *d*	*M*	*SD*	*M*	*SD*
Scientific Reasoning	3.36	0.001	0.42	2.09	1.72	2.79	1.64

**Table 6.** Independent Sample t-Test results for student experimental design skills

				Pretest		Posttest	
Variable	*t*(276)	*p*	Cohen’s *d*	*M*	*SD*	*M*	*SD*
Experimental Design	2.70	0.007	0.34	2.57	1.14	2.94	1.07

**Table 7.** Results for multiple linear regression examining how skills in experimental design, scientific reasoning, and scientific research affect possible scientific selves

Source	*B*	*SE*	β	*t*	*p*
Experimental Design	0.06	0.04	.09	1.42	.156
Scientific Research Skills	0.35	0.06	.33	5.55	.001
Scientific Reasoning	0.06	0.03	.13	2.19	.030

*Note. F*(3,271) = 22.88, *p* < .001, *R*^*2*^ = 0.20

## APPENDIX A

The 8 Practices of the *Next Generation Science Standards*

1. Asking questions (for science) and defining problems (for engineering)
2. Developing and using models
3. Planning and carrying out investigations
4. Analyzing and interpreting data
5. Using mathematics and computational thinking
6. Constructing explanations (for science) and designing solutions (for engineering)
7. Engaging in argument from evidence
8. Obtaining, evaluating, and communicating information
